# *Case of* Fracture *Cured by the Use of Nitric Acid*

**Published:** 1805-07-01

**Authors:** 

**Affiliations:** Abbeville


### *Case of* Fracture *cured by the Use of Nitric Acid, by Dr.* Penel *of Abbeville.*

An elderly man, whose left thigh had been fractured obliquely, at its inferior part, was received into the Civil and Military Hospital at Abbeville. The reduction of the fractured bone was easily obtained, and the symptoms soon disappeared. On the fourteenth day, when the dressings were removed, the parts appeared in a good state; the following morning, however, the patient complained very much of his thigh, although the fracture seemed not to be deranged. On the nineteenth day the bone was slightly swollen; the callus appeared to get more consistency; but the fever continued. On the twenty-seventh day, Dr. Penel, having removed the dressing, perceived more evidently the swelling of the bone; and the callus exhibiting some inequalities, caused him to suspect the presence of a morbid poison. About this time, the Peruvian bark and antiscorbutic remedies were employed, but without the intended success. On the fortieth day the bone appeared still more tumid; the callus presented a considerable tumour, which appeared solid. The dressings were now entirely removed, and the limb laid on a bolster; after which the patient found himself better; but, on a sudden, the muscles contracted, the callus was destroyed, and the whole member shortened; and it was necessary to make a forced and lasting extension, in order to reduce it to its former length. Dr. Penel observing that the urine deposited a considersble quantity of a greenish substance, found it, on examination, to be phosphat of lime. The swelling of the limb disappeared a few days after, and the fracture seemed again to consolidate; the urine, however, remained the same, and was passed in a greater quantity; the febrile motions continued, but the swelling of the bone was not augmented. Two months after the fracture, the callus appeared again solid, and Dr. Penel continued the dressings twenty days longer; but they were scarcely removed, when the patient found himself unable to stand, and perceived the same symptoms as at first. On visiting him, two days after, the extremities of the fracture separated, and the author was obliged to undertake the reduction a third time. He now determined to give the patient, internally, nitric acid, which he accordingly took, in a dose of one demi gramme per day (about ℈ß) diluted in a kilogramme of water, (about ℔ii). The urine became clearer, the fever ceased, and, eight days afterwards, the patient found himself so well, that he desired the dressings might be removed; this request, however, was not complied with, till the fourth month after his first reception, when he left the hospital, as he could walk with great ease, and without crutches.

